# Prevalence of Cysticercosis in Pigs and Risk Assessment Among Occupationally Exposed Workers in Benue State, Nigeria

**DOI:** 10.1002/vms3.70367

**Published:** 2025-04-22

**Authors:** Alex A. Adikwu, Tyonginengen Iorparegh, Felix M. Per, Andrew M. Adamu, Olajide A. Owolodun, Paul F. Horwood, Theophilus I. Emeto, Oyelola A. Adegboye

**Affiliations:** ^1^ Department of Veterinary Public Health and Preventive Medicine College of Veterinary Medicine University of Agriculture Makurdi Nigeria; ^2^ College of Public Health Medical and Veterinary Sciences James Cook University Townsville QLD Australia; ^3^ Australian Institute of Tropical Health and Medicine Building 48 James Cook University Townsville QLD Australia; ^4^ Biotechnology Division National Veterinary Research Institute Vom Nigeria; ^5^ World Health Organization Collaborating Centre for Vector‐Borne and Neglected Tropical Diseases College of Public Health Medical and Veterinary Sciences James Cook University Townsville QLD Australia; ^6^ Menzies School of Health Research Charles Darwin University Darwin Australia

**Keywords:** cysticercosis, one health, pigs, prevalence, taenia solium, zoonoses

## Abstract

**Aim:**

Cysticercosis is a key zoonotic disease burden, posing significant public health challenges. Neurocysticercosis, a sequela associated with the larval stage of *Taenia solium*, is a recognised cause of late‐onset epilepsy in humans, thereby highlighting the need for robust epidemiological data. This study investigated the prevalence of cysticercosis in slaughtered pigs within Makurdi, Benue State, Nigeria, to contribute to understanding the disease's local epidemiology and associated risk factors.

**Methods:**

From January to August 2019, we examined pig carcasses at four abattoirs: Utyondo market, Wurukum abattoir, Modern market, and Railway abattoirs. Structured questionnaires were administered to butchers, pig farmers, and civil servants to gather information on disease knowledge. A total of 2,176 pig carcasses were examined.

**Results:**

Among the examined carcasses, 24 (1.1%) were infected. Of these, 18 (0.83%) were boars, and 6 (0.28%) were sows. The highest prevalence was observed in Utyondo market (2.75%), followed by Wurukum (0.77%) and Railway markets (0.65%). The diaphragm (odds ratio (OR) = 0.09; 95% confidence interval (CI): 0.01‐0.70) and the tongue (OR = 0.18; 95% CI: 0.04‐0.81) were less likely to be infected compared to the shoulder. Knowledge of cysticercosis was relatively high across all participants (71.72%, 95% CI: 61.77‐80.31).

**Conclusion:**

Our findings indicate the endemicity of porcine cysticercosis in Makurdi abattoirs. While knowledge of the disease seems relatively widespread among exposed populations, gaps exist in understanding transmission routes. A collaborative One Health approach involving studies on occupationally exposed individuals is recommended for a comprehensive understanding of the disease burden and to develop targeted control strategies.

## Graphical abstract

• Porcine cysticercosis is endemic in pigs raised in Makurdi slaughterhouses. The prevalence is observed to be higher in older pigs, suggesting that age may be a potential risk factor.

• Cysticerci were predominantly found in the shoulder muscles of pig carcasses, highlighting the part as a critical focus for future interventions.

• Workers' occupational exposure, coupled with their substantial knowledge yet partial awareness of the zoonotic potential, suggests the urgent need for comprehensive educational programmes.

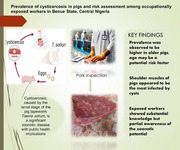

## 1 Introduction

*Taenia solium*, a zoonotic cestode parasite, represents a major public health and economic burden globally, with a disproportionate impact on resource‐limited settings (Dixon et al. 2022). Endemic regions include sub‐Saharan Africa, Central Asia, and Latin America, where transmission is facilitated by free‐ranging pig production, inadequate sanitation, and deficient meat inspection practices (Weka et al. 2020). The larval stage (cysticercus) causes cysticercosis, occurring when pigs or humans ingest *T. solium* eggs through contaminated food, water, or undercooked infected pork (Poudel et al. 2019). Following ingestion, eggs hatch in the small intestine, and the larvae disseminate, forming cysts in various tissues, including muscles and the central nervous system.

Several risk factors contribute to the disease dissemination of cysticercus, including low socioeconomic status (Wardrop et al., 2021), poor hygiene or eating habits, and suboptimal livestock management practices (Adenuga et al., 2018; Adesokan and Adeoye, 2019). In pigs, cysticercosis manifests with clinical signs such as diarrhoea, myositis, emaciation, myocardial failure, abnormal skin sensitivity, seizures, and neurological disorders resulting in significant economic losses due to carcass condemnation and reduced productivity (Adenuga et al. 2017; Widdowson et al. 2000).

The emergence of cysticercosis in previously non‐endemic regions driven by globalisation and increased travel highlights the ongoing challenge posed by the parasite (Lightowlers et al. 2016). Cysticercosis, particularly neurocysticercosis, contributes substantially to disability and lost productivity, with an estimated 2.8 million disability‐adjusted life‐years lost annually in endemic regions (Havelaar et al. 2015). Sub‐Saharan Africa is particularly vulnerable due to the prevalence of factors that favour the parasite's life cycle (Ngowi et al. 2010). Cysticercosis represents an important health and socio‐economic burden on pig production in many African countries (Assana et al. 2019). Prevalence studies in pigs across Africa have reported varying rates ranging from 5% to 33% (Gulelat et al. 2022).

Nigeria's rapidly expanding pig population increasing from 865,000 heads in 1971 to 7.99 M heads in 2020 (Knoema 2022) is driven by rising pork demand. Pigs are predominantly reared in semi‐intensive systems characterised by close human‐animal interaction. (Bata et al. 2020; Adamu et al. 2024). Despite the economic importance of pig production and the public health threat posed by cysticercosis, information regarding its prevalence in Nigeria remains limited. Existing studies report a broad range of prevalence rates, suggesting a potential underestimation of the true burden (Onah and Chiejina, 1995; Karshima et al., 2013; Adesokan and Adeoye, 2019).

This study aimed to address the knowledge gap regarding *T. solium* cysticercosis in Nigeria by estimating the prevalence of cysticercosis in pigs slaughtered at selected abattoirs in Makurdi, Benue State. Additionally, we assessed the knowledge and awareness of cysticercosis among occupationally exposed individuals, including butchers, pig farmers, and civil servants. Understanding the prevalence and level of awareness is crucial for developing effective control strategies to safeguard public health and minimise economic losses associated with cysticercosis in Nigeria's pig industry.

## 2 Methods

### 2.1 Study Design and Setting

**FIGURE 1:**
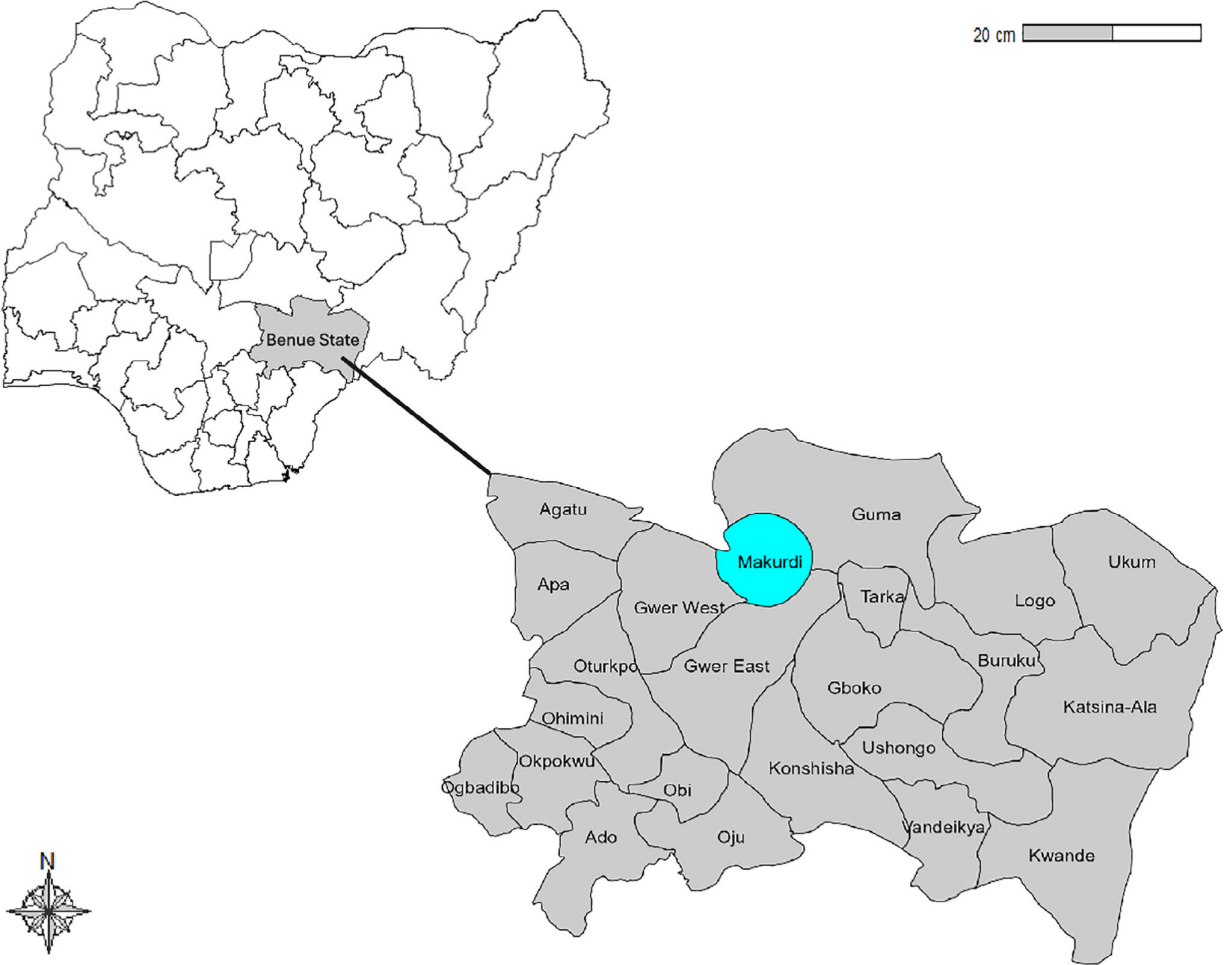
Map of Nigeria and Benue State showing the study area in Makurdi.

This cross‐sectional study was conducted from January to August 2019 in Makurdi, the capital of Benue State, Nigeria (Figure 1). Makurdi lies along the Benue Riverbank with a population of approximately 471,754 individuals (United Nations World Urbanization Prospects, 2024). Four major slaughterhouses (Wurukum, Railway, Modern Market, and Utyondo) were selected for pig examination and data collection.

### 2.2 Sampling and Identification

Convenience sampling was adopted. Sample sites were visited three times a week during the study period. Data on the number of slaughtered pigs, age, sex, and slaughterhouse location were recorded.

### 2.3 Post‐Mortem Examination

A total of 2176 pig carcasses were inspected using a combination of palpation and deep incision techniques as described by examined organs included the heart, diaphragm, masseter muscles, tongue, neck, shoulder, intercostal and abdominal muscles.

### 2.4 Cyst Identification

Suspected *T. solium* cysts were identified based on translucence with visible scolices or bluish‐green caseous masses. These lesions were excised, placed in flasks with ice packs, labelled, and transported to the laboratory for confirmation. Confirmation involved morphological examination with the naked eye and an optical stereomicroscope (S/ST series, Olympus Optical Co. Ltd., Tokyo, Japan). *T. solium* cysticerci was identified based on characteristic features, including oval shape (approximately 10 mm x 5 mm or larger), translucent whitish parasite membrane, and visible scolex within a fluid‐filled cyst.

### 2.5 Knowledge, Attitudes and Practices (KAP) Assessment

#### 2.5.1 Questionnaire Development

A pre‐tested, closed‐ended questionnaire was used to assess knowledge, attitudes, and practices (KAP) related to cysticercosis among pig farmers, butchers and civil servants (meat inspectors, abattoir workers and meat traders). After informed consent was obtained in accordance with the Helsinki Declaration (2001), participants completed the questionnaire. Content validity was established through cross‐referencing (Cronbach's alpha = 0.7).

#### 2.5.2 Questionnaire Content

The questionnaire comprised four sections. The first section gathered basic information and qualifications. Sections two and three focused on hygiene practices, pork consumption habits, and activities potentially contributing to cysticercosis transmission. The final section assessed knowledge and awareness of cysticercosis characteristics, transmission routes, prevention, and control measures. Information collected included:

- Pork consumption habits
- Hygiene practices during pork handling and cooking
- Waste disposal methods and facilities
- Knowledge of cysticercosis
- Awareness of disease characteristics, transmission, prevention and control

### 2.6 Data Analysis

Microsoft Excel was used for data entry and management. Subsequently, data was exported to SPSS version 25 (IBM) and R version 4.3.1 for analysis. Descriptive statistics were employed to summarise participant demographics, KAP responses, and prevalence of cysticercosis. Percentages were reported with corresponding 95% confidence intervals calculated using the Clopper‐Pearson method. A binary logistic regression (generalised linear model) was conducted to assess the relationship between cysticercosis occurrence and variables like sex, location, age and specific pig organs. Odds ratios with 95% confidence intervals (CI) were reported. All statistical tests were two‐tailed with a significance level of *p* < 0.05.

## 3 Results

### 3.1 Prevalence of *T. solium* Amongst Pigs

A total of 2,176 carcasses were examined from four slaughterhouses, with the majority of animals sampled being boars (72.5%). *T. solium* infection was detected in 18/1577 (1.14%, 95% CI: 0.68, 1.8) boars and 6/599 (1.0%, 95% CI: 0.37, 2.17) sows, with an overall prevalence of 1.1% (95% CI: 0.71, 1.64). Pigs 6–10 months old were the most presented for slaughter. However, the infection was least prevalent in this group (0.54%, 95% CI: 0.20, 1.16). The highest prevalence (7.14%) was detected in older pigs between 21and 25 months. Amongst the five tissues examined, the shoulder presented the highest level of infection (0.51%, n = 11), followed by the neck (0.28%, n = 6), masseter (0.18%, n = 4), tongue (0.09%, n = 2) and the diaphragm (0.05%, n = 1). In Utyondo market slaughterhouse, 15 out of 545 (2.75%) carcasses were positive for cysticercosis. Wurukum market had the highest number of carcasses inspected (36.1%) with a prevalence of 0.65%, while a prevalence of 0.77% was detected in Modern market, and cysticercosis was not detected in any of the animals at Railway market (Table 1).

**TABLE 1:** Prevalence and risk of *T. solium* in pigs.

	n/N	% (95%CI)	P‐value	OR (95%CI)
Overall	24/2,176	1.1 (0.71, 1.64)		
Sex				
Male	18/1,577	1.14(0.68, 1.80)	Ref	Ref
Female	6/599	1.00(0.37, 2.17)	0.78	0.88 (0.35, 2.22)
Age (months)				
0−5	2/206	0.97(0.12, 3.46)	Ref	Ref
6−10	6/1,121	0.54(0.20, 1.16)	0.45	0.55 (0.11, 2.74)
11−15	9/643	1.40(0.64, 2.64)	0.64	1.44 (0.31, 6.76)
16−20	4/164	2.44(0.67, 6.13)	0.27	2.55 (0.46, 14.10)
21−25	3/42	7.14(1.50,19.48)	**0.01** *	7.8 (1.27, 48.53)
Organs				
Shoulder	11/2,176	0.51 (0.25, 0.90)	Ref	Ref
Neck	6/2,176	0.28 (0.10, 0.59)	0.22	0.54 (0.20, 1.47)
Tongue	2/2,176	0.09 (0.01, 0.33)	**0.01** *	0.18 (0.04, 0.81)
Masseter	4/2,176	0.18 (0.05, 0.47)	0.07	0.36 (0.12, 1.14)
Diaphragm	1/2,176	0.05 (0.00, 0.26)	**0.004** *	0.09 (0.01, 0.70)
Site				
Utyondo market	15/545	2.75 (1.55, 4.50)	Ref	Ref
Wurukum market	5/768	0.65 (0.21, 1.51)	**0.0022** *	0.23 (0.08, 0.64)
Modern market	4/522	0.77 (0.21, 1.95)	**0.014** *	0.27 (0.09, 0.83)
Railway market	0/341			

*Bold: Significance.

### 3.2 Risk Factors of *T. solium* Infection in Pigs

Statistical comparison of *T. solium* infections and pig characteristics showed a significant association with age, organs, and site (Table 1). The risk of cysticercosis in male carcasses was slightly higher compared to females, but it was not statistically significant when compared. The risk of *T. solium* infection in pigs 21–25 months old was significantly higher than those in the 0–5 category (OR = 7.8; 95% CI: 1.27‐48.53). The risk of infection was lower in the diaphragm (OR = 0.09; 95% CI: 0.01‐0.70) and the tongue (OR = 0.18; 95% CI: 0.04‐0.81) than in the shoulder region. Compared to pig carcasses examined in Utyondo market, carcasses examined in Wurukum and Modern markets were less likely to be infected with cysticercosis, with ORs of 0.23 (95%CI: 0.08‐0.64) and 0.27 (95% CI: 0.09‐0.83), respectively.

### 3.3 Characteristics of Respondents

One hundred individuals, including butchers, pig farmers, and civil servants with varying levels of education, responded to the questionnaire. 74% were males, 62% were between the ages of 21 to 40 years, 67 (69.07%) had undertaken a tertiary education, and only one (1.03%) respondent had no formal education. Of the 97 respondents that agreed to disclose their educational level, 75.2% were civil servants, 14.4% were butchers, and 10.3% were pig farmers (Table 2). Most respondents emerged from North Bank (36/100), while Kanshio recorded the least (6/100).

**TABLE 2:** Characteristics of respondents in the study (n = 100).

Variable	n (%)
**Gender**	
Male	74 (74%)
Female	26 (26%)
**Age (years)**	
<20	4 (4%)
21–40	62 (62%)
41− 60	27 (27%)
>60	7 (7%)
**Educational Level** *	
No formal education	1 (1.03%)
Primary	6 (6.19%)
Secondary	23 (23.71%)
Post‐Secondary (Tertiary)	67 (69.07%)
**Primary activity** *	
Butcher	14 (14.43%)
Civil servant	73 (75.26%)
Pig farmer **residence**	10 (10.31%)
Wurkum High level North bank Kanshio Modern market	19 (19%) 23 (23%) 36 (36%) 6 (6%) 16 (16%)

*Three participants did not respond to this question.

### 3.4 KAP of Occupationally Exposed Workers

Despite the high number of respondents (71.72%, 95% CI: 61.77‐80.31) who reported that they were knowledgeable about porcine cysticercosis, 48 (48.49%) were unaware that it is zoonotic, and 33.7% (95% CI: 24.03‐44.51) were unsure of the mode of transmission. Twenty‐seven (30.34%, 95% CI: 21.03‐40.99) respondents opined that eating half‐cooked pork (medium rare) causes the disease, which was higher than those who attributed eating raw cabbage as the mode of transmission (5.62%, 95% CI: 1.85‐12.63). In assessing preventive measures, 20.43% (95% CI: 12.77‐30.05) of the respondents proposed avoiding half‐cooked pork, while 8.6% (95% CI: 3.79‐16.24) suggested halting meat consumption. However, maintaining good hygiene was the preferred method of preventing the disease (37.63%, 95% CI: 27.79‐48.28) compared to the other listed measures suggested, and 75.32% (95% CI:64.18‐84.44) of respondents agreed that adequately cooked pork is a better preventive measure for cysticercosis than roasted pork (medium) (16.88%, 95% CI: 9.31‐27.14) and half‐cooked pork. In addition, a high number of respondents were in support of meat inspection (82.35%, 95% CI:72.57‐ 89.77) as a preventive measure for disease transmission rather than banning meat (12.94%, 95% CI: 6.64‐21.98) or discontinuing pig farming (4.71%, 95% CI: 1.30‐11.61) (Table 3). 75 respondents (78.13%, 95% CI:72.57‐89.77) were willing to present themselves for mass screening, while 84 (85.71%, 95% CI: 77.19‐91.96) respondents would accept medications in the event of a mass intervention program. (Table 3)

**TABLE 3:** Responses towards *Porcine Cysticercosis*, transmission, hygiene and prevention.

Variable	Responses	95% CI
Do you eat pork?	*n = 100*	
Yes	79 (79%)	69.71‐86.51
No	21 (21%)	13.50‐30.29
Method of consumption	*n = 77*	
Adequately cook	58 (75.32%)	64.18‐84.44
Roasted (Medium)	13 (16.88%)	9.31‐27.14
Raw	0 (0.0%)	
Half‐cooked (Medium rare)	6 (7.79%)	2.91‐16.19
Have you heard of Cysticercosis?	*n = 99*	
Yes	71 (71.72%)	61.77‐80.31
No	28 (28.28%)	19.69‐38.22
Cysticercosis causes disease?	*n = 99*	
Yes	51 (51.51%)	41.25‐61.68
No	48 (48.49%)	38.32‐58.75
Mode of infection	*n = 89*	
Eating half‐cooked pork	27 (30.34%)	21.03‐40.99
Poor hygiene	27 (30.34%)	21.03‐40.99
Eating raw cabbage	5 (5.62%)	1.85‐12.63
Not sure	30 (33.70%)	24.03‐44.51
Preventive method	*n = 93*	
Avoid eating cabbage and salad	9 (9.68%)	4.52‐17.58
Maintain good hygiene	35 (37.63%)	27.79‐48.28
Do not eat any meat	8 (8.60%)	3.79‐16.24
Avoid eating half‐cooked pork	19 (20.43%)	12.77‐30.05
Not sure	22 (23.66%)	15.46‐33.60
Prevention of transmission	*n = 85*	
Meat inspection	70 (82.35%)	72.57‐89.77
Banning the use of poor meats	11 (12.94%)	6.64‐21.98
Disallowing pig farming	4 (4.71%)	1.30‐11.61
Waste disposal method	*n = 99*	
Open defecation	9 (9.10%)	4.24‐16.56
Bush	5 (5.05%)	1.65‐11.39
Water system	78 (78.79%)	69.42‐86.36
Pit latrine	7 (7.07%)	2.89‐14.03
Mass screening participation?	*n = 96*	
Yes	75 (78.13%)	68.53‐85.92
No	24 (21.87%)	16.72‐34.88
Accept drugs for mass intervention?	*n = 98*	
Yes	84 (85.71%)	77.19‐91.96
No	14 (14.29%)	8.04‐22.81

## 4 Discussion

This study, conducted across four abattoirs in Makurdi, revealed a *T. solium* prevalence of 1.1% in pigs as determined by necropsy and microscopic examination, confirming the presence of porcine cysticercosis within the region. This prevalence is notably lower than previously reported in other Nigerian locales including Ibadan (4.4%, Adesokan et al., 2019), Jalingo (4.95%, Agere et al., 2016), and Zuru (14.4%, Gweba et al., 2010). Although a marginally higher prevalence was observed in boars (1.14%) compared to sows (1%), no statistically significant sex‐based risk for infection was identified. This observation aligns with several previous studies (Karshima et al. 2013; Agere et al. 2016) that similarly reported a lack of significant association between sex and cysticercosis. Conversely, age exhibited a correlation with cysticercosis prevalence, particularly in older pigs (21‐25 months). This finding diverges from studies by Weka et al. (2009), Secka et al. (2010), Poudal et al. (2018), and Adesokan et al. (2019), which reported no discernible age‐related differences. While our findings share similarities with those of Gweba et al., 2010; Pondja et al., 2010; and Agere et al., 2016) regarding the presence of cysticercosis across various age groups, the absence of data regarding the management systems of younger and older pigs necessitates further investigation to validate age as a potential risk factor.

Interestingly, the highest cyst concentrations were identified in the shoulder, neck, and masseter muscles. These parts, often overlooked during routine meat inspections, may contribute to the preservation of carcass value, as attention as primary inspection efforts are typically focused on internal organs. This observation reinforces findings from previous predilection studies (Boa et al., 2001; Biu et al., 2012; Lightowlers et al., 2015; Kabululu et al., 2020) and underscores the patterns associated with extensive farming practices, as observed in samples from Utyondo market. Furthermore, this survey highlighted evolving gender dynamics within the meat‐handling sector, with a notable increase in female participation (26%) in a traditionally male‐dominated field (Eshitera et al. 2012; Nyangi et al. 2022). This demographic shift necessitates the implementation of inclusive control strategies that effectively engage female stakeholders.

Despite the relatively high level of education among participants, which may have contributed to increased awareness of cysticercosis and its zoonotic implications, only 30% of participants recognised the risks associated with half‐cooked pork and inadequate hygiene practices. Persistent unsafe defecation habits among some individuals pose a potential risk factor for continued transmission (Pouedet et al., 2002; Shey‐Njila et al., 2003; Ngowi et al. 2004).

While most respondents advocated for stringent hygiene measures as a preventive strategy, identified knowledge gaps and diverse opinions regarding meat inspection and farm regulations highlight the necessity for intensified public health education initiatives. The observed willingness to participate in screening and treatment programmes during mass interventions indicates a positive community receptiveness to health‐related initiatives.

The lower prevalence observed in our study may be attributed to several factors. First, procedural interruptions and impatience from butchers and animal owners during organ incisions could have potentially compromised the detection accuracy. Animal owners often express concern that incisions reduce the market value of the organs, particularly given the uncertainty of government compensation. Second, the majority of pigs examined were raised under semi‐intensive management systems, which likely reduce exposure to infections compared to small‐holder farms and free‐ranging systems, as reported by Gweba et al. (2010) and (Kungu et al. 2017). Furthermore, variations in prevalence may reflect the impact of enhanced public health campaigns and educational initiatives such as those promoted during the World Neglected Tropical Diseases Day. Finally, regional differences in cysticercosis prevalence may stem from unidentified factors requiring further investigation.

## 5 Conclusions

This study confirms the endemicity of porcine cysticercosis in pigs slaughtered at Makurdi abattoirs, with a notable prevalence observed in older pigs. Further studies examining pig origin and management practices are recommended to validate age as a potential risk factor for cysticercosis. Cysticerci were predominantly located in the shoulder muscles, and the Utyondo market exhibited the highest number of cases, signifying its critical role for future intervention strategies. These programmes should aim to improve understanding of *T. solium* transmission among high‐risk populations and emphasise the importance of thorough cooking and improved sanitation to mitigate this public health threat. Further epidemiological studies are necessary to refine control strategies and effectively reduce the prevalence of this neglected tropical disease.

## Author Contributions

**Alex Adikwu**: conceptualisation, formal analysis, investigation, methodology, project administration, resources, supervision, validation, visualization, writing – original draft, writing–review and editing. **Tyonginengen Iorparegh**: investigation, methodology and project administration. **Felix Per**: methodology and project administration. **Adamu Andrew**: conceptualisation, investigation, methodology, validation, writing–review and editing. **Olajide Owolodun**: conceptualisation, investigation, methodology, validation, writing–review and editing. **Paul Horwood**: conceptualisation, investigation, methodology, validation, writing–review and editing. **Theophilus Emeto**: conceptualisation, investigation, methodology, validation, writing–review and editing. **Adegboye Oyelola**: formal analysis, methodology, visualisation, writing–review and editing.

## Ethics Statement

Ethical approval was obtained from the Animal Ethics Committee of the Department of Livestock Services, Ministry of Agriculture and Natural Resources, Benue State, Nigeria (MANR/VET/RES/258). Verbal informed consent was obtained from respondents prior to sample collection and questionnaire administration.

## Conflicts of Interest

The authors declare no conflict of interest.

## Data Availability

Data is available on request.
